# *miR-7847-3p* Serves as a Prognostic Biomarker and Suppresses Colorectal Cancer Progression

**DOI:** 10.5152/tjg.2026.25349

**Published:** 2026-01-05

**Authors:** Haiqin Chen, Yusen Wang, Ming Lu

**Affiliations:** 1 Department of Anal Medicine, The First Affiliated Hospital of Xinjiang Medical University, Urumqi, China; 2 Department of Gastrointestinal Surgery, The First Affiliated Hospital of Xinjiang Medical University, Urumqi, China

**Keywords:** *AP1S1*, colorectal cancer, *miR-7847-3p*, prognostic biomarkers, tumor suppression

## Abstract

**Background/Aims:**

*MiR-7847-3p* is aberrantly expressed in multiple cancer types; however, its biological function and molecular mechanism in colorectal cancer (CRC) remain unclear. This investigation aims to explore the function of *miR-7847-3p* in CRC development and its potential regulatory mechanisms.

**Materials and Methods:**

Tumor tissues and paracancerous normal tissues from 122 CRC patients were collected and *miR-7847-3p* expression was detected by quantitative reverse transcription polymerase chain reaction. The predictive capacity of *miR-7847-3p* was evaluated through Kaplan–Meier survival analysis and subsequently validated via multivariable Cox proportional hazards modeling. The effects of *miR-7847-3p* on HCT116 and SW480 cells were evaluated through Cell Counting Kit-8 and Transwell assays. The *miR-7847-3p* target genes were predicted using TargetScan, miRDB, and miRWalk. Dual-luciferase reporter assay confirmed the regulatory relationship between *miR-7847-3p* and *AP1S1*.

**Results:**

The *miR-7847-3p* expression was downregulated in CRC tumor tissues and CRC cell lines (HCT116 and SW480 cells). Reduced expression levels of *miR-7847-3p* are associated with advanced lymph node metastasis, Tumor-node-metastasis stage, and poorer 5-year survival. The *miR-7847-3p* downregulation signature proved to be a standalone prognostic indicator in CRC. Functional studies indicated that overexpression of *miR-7847-3p* inhibited CRC cell proliferation, migration, and invasion, while its knockdown promoted these malignant behaviors. Bioinformatics predictions and dual luciferase reporter assay confirmed *AP1S1* is a downstream gene of *miR-7847-3p*.

**Conclusion:**

*MiR-7847-3p* is downregulated in CRC and is associated with poor prognosis. Its overexpression can inhibit the proliferation, migration, and invasion of CRC cells, possibly by targeting *AP1S1*, suggesting the dual potential of *miR-7847-3p* as a prognostic biomarker and therapeutic target.

**Main Points**

- Downregulated *miR-7847-3p* serves as an independent poor prognostic factor in colorectal cancer (CRC).
- Overexpression of *miR-7847-3p* inhibited the malignant behaviors of CRC cells.
- *MiR-7847-3p* functionally interacts with* AP1S1.*

## Introduction

Colorectal cancer (CRC) is a common malignant tumor, with a worldwide incidence of 1 926 000 and mortality of 904 000 in 2022. This malignancy accounted for nearly one-tenth of all cancer cases (9.6%) and deaths (9.3%).^1^ Despite the current clinical use of colonoscopy combined with imaging assessment as the primary diagnostic tool, there is a high rate of underdiagnosis of early lesions^2^ and a lack of efficient molecular markers for prognostic stratification. Conventional treatment methods (such as surgical resection and chemotherapy) have limited efficacy for metastatic CRC, resulting in significantly reduced 5-year survival rates (less than 15%), increased risk of tumor recurrence, and severe treatment side effects (such as bone marrow suppression and intestinal dysfunction).^3^^,^^4^ The current clinical landscape demands rapid advancement in biomarker discovery to enable precise diagnosis and personalized treatment, thereby improving patient outcomes.

MicroRNAs (miRNAs) are involved in tumor development by regulating gene expression networks, and these molecular aberrations are pathologically implicated in driving tumor proliferation, metastatic spread, and chemoresistance development in CRC.^5^ The exceptional stability of miRNAs in bodily fluids and their tissue-specific expression patterns have positioned them as promising diagnostic biomarkers.^6^^-^^8^ Although some miRNAs have shown diagnostic potential in clinical studies on CRC,^9^^-^^12^ the number of miRNA markers translated into clinical applications remains limited. This underscores the urgent need for deeper research to discover and validate miRNA-based diagnostic markers and therapeutic targets. With the advancement of miRNA detection technology^13^ and the establishment of standardized sample processing processes,^14^ the reliability of miRNA marker research has been significantly improved. Based on the above technical advantages and clinical needs, screening miRNAs abnormally expressed in CRC is of great value for improving the disease diagnosis and treatment system.^15^

The research of *miR-7847-3p* in the field of oncology has gradually received attention. One study reported that *miR-7847-3p* was clearly downregulated in serum exosomes of patients with sepsis,^16^ and its expression was lower in bladder cancer tissues and shows an inverse relationship with tumor stage.^17^ Regarding CRC, only 1 microarray analysis study based on miRNA from CRC patient serum exosomes has reported downregulation of *miR-7847-3p*.^18^ However, the expression characteristics of *miR-7847-3p* in CRC, its clinical pathological relevance, and its regulatory mechanisms remain to be elucidated.

Given the key regulatory function of *miR-7847-3p* in cancers, investigating the clinical significance of *miR-7847-3p* in CRC will help deepen the understanding of CRC pathogenesis. This research endeavors to uncover the function and mechanisms of *miR-7847-3p* in CRC, thereby providing new potential molecular markers and a theoretical basis for the treatment of CRC.

## Materials And Methods

### Patients and Specimens

Tumor specimens and matched histologically normal adjacent tissues (sampled at a distance of ≥5 cm from the neoplastic edge^19^ were obtained from 122 CRC patients treated between January 2018 and December 2020. The specimens were independently verified by 2 pathologists. The study protocol was approved by the Ethics Committee at the First Affiliated Hospital of Xinjiang Medical University (approval number: 2018003, date: January 10, 2018), and this study adhere to the tenets of the Declaration of Helsinki. All enrolled subjects providing documented consent. Cases involving preoperative radiation therapy or concurrent additional malignancies were excluded from the analysis. The outcomes of all patients were monitored by telephone follow-up after surgery for 5 years. The postoperative treatment plans for the patients were all meticulously documented. For patients with stage II and III CRC, adjuvant chemotherapy regimens such as capecitabine combined with oxaliplatin (CapeOX) or fluorouracil, leucovorin, and oxaliplatin are employed. For metastatic CRC patients with RAS wild-type tumors, targeted therapies such as cetuximab or bevacizumab are used for treatment. For CRC patients with mismatch repair deficiency or high microsatellite instability, the use of immune checkpoint inhibitors such as pembrolizumab for immunotherapy will be explored. The specific treatment plan will be comprehensively evaluated based on factors such as tumor stage and the patient’s overall condition and will be implemented under the guidance of a multidisciplinary team.

### Cell Culture

Colon cancer cells (SW480 and HCT116 cells) and normal control cells (CCD841) were sourced from the American Type Culture Collection. These cells were cultured in RPMI-1640 medium with 10% fetal bovine serum at 37°C and 5% CO_2_.

### Cell Transfection

The transfection complexes were prepared by incubating either *miR-7847-3p* mimic or inhibitor (Reebok Bio) with Lipofectamine 3000 at a 1 : 10 (v/v) ratio in SW480 and HCT116 cell lines and cultured with Opti-MEM medium. Both cell lines were transfected with *miR-7847-3p* mimic complexes to induce *miR-7847-3p* overexpression, while loss-of-function experiments utilized inhibitor complexes for* miR-7847-3p* knockdown. Both mimic NC and inhibitor NC were parallelly transfected as the negative controls. Cells were harvested at the 48-hour timepoint following transfection for downstream analyses.

### Reverse Transcription Quantitative Polymerase Chain Reaction Detection

Total RNA was extracted with Trizol (Invitrogen, USA). Total RNA (1 μg) was reverse transcribed into miRNA (Mir-X™ miRNA First-Strand Synthesis Kit, Takara) and mRNA (PrimeScript™ RT Reagent Kit, Takara). The cDNA was then subjected to quantitative polymerase chain reaction (qPCR) for amplification and detection. Primer sequences: *miR-7847-3p* (forward: 5′-CGTGACTGTCCCTCTGTGTC-3′, reverse: universal primer in Mir-X Kit); *U6* (forward: 5′-CTCGCTTCGGCAGCACA-3′, reverse: 5′-TGCGTGTCATCCTTGCGCAG-3′); *GAPDH* (forward: 5′-GAAGGTGAAGGTCGGGAGTC-3′, reverse: 5′-GAAGATGGTGATGGGATTTC-3′); *AP1S1* (forward: 5′-GCTGGAGGAGGAGGTGGAAGAGA-3′, reverse: 5′-CAGGTAGGCGTTGTCCTTGT-3′). Expression levels were calculated using the 2^-ΔΔCT^ method, with normalization to *U6* (miRNA analyses) and *GAPDH* (mRNA analyses) as internal controls. In this study, *U6* and *GAPDH* were selected as reference genes for miRNA and mRNA analyses, respectively. The stability of these reference genes was validated using geNorm and NormFinder software, which showed good stability (M value < 0.5) across different sample groups. Additionally, preliminary screening of reference gene expression levels ensured stable expression in all samples.

### Cell Counting Kit-8

To assess cellular proliferation, a 96-well plate (5 × 10^3^ cells per well) was inoculated with 5 × 10^3^ cells per well and cultured for varying durations (0, 24, 48, and 72 hours). At each designated interval, 10 μL of Cell Counting Kit-8 (CCK-8) reagent (Nippon Kohin Chemical) was introduced, followed by a 2-hour incubation at 37°C. Optical density measurements were subsequently obtained at 450 nm to quantify viable cells. This experimental procedure was conducted in triplicate to ensure reproducibility.

### Transwell

Cell migration: transfected cells (5 × 10^4^ cells/chamber) were resuspended in a serum-free medium and inoculated into the upper chamber of Transwell. Complete medium containing 10% fetal bovine serum (FBS) (600 μL) was added to the lower chamber as a chemokine. Following 24 hours incubation at 37°C, non-migrated cells on the membrane’s upper surface were gently removed. Cells were fixed, stained with crystal violet, and counted in 5 random fields.

Cell invasion: Firstly, Matrigel (BD Biosciences, NY, USA) was diluted with serum-free medium in the ratio of 1 : 8, evenly encapsulated on the membrane surface of the upper chamber of Transwell (Corning, NY, USA), and incubated at 37°C for 4 hours to form a gel layer. Transfectants (1 × 10^5^ cells/chamber) were suspended in serum-deprived medium and plated in the upper compartment. The lower compartment contained 400 μL of basal medium with 10% FBS as a chemoattractant. After 48 hours of incubation at 37°C, uninvaded cells were removed as above, fixed, stained, and counted for invaded cells crossing the stromal gel layer.

### RNA Immunoprecipitation

RNA immunoprecipitation experiments were performed using the Magna RIP™ system (Millipore). The anti-Ago2 antibody (Abcam) and homologous IgG were incubated with cell lysate at 4°C overnight. The RNA was extracted after the Protein A/G magnetic bead capture complex, and the *miR-7847-3p* enrichment was detected by qPCR. The IgG group was used as a negative control.

### Bioinformatics Analysis

The targets of *miR-7847-3p* were predicted using bioinformatics analysis through the TargetScan, miRDB, and miRWalk databases. Consensus targets identified by the Venn diagram from all 3 databases were selected for further analysis.

### Dual-luciferase Assay

The 3′UTR of *AP1S1* (WT) was amplified and cloned into the pmirGLO vector (Promega). A mutant (MT) version of the *AP1S1* 3′UTR, with predicted *miR-7847-3p* binding sites disrupted, was created via site-directed mutagenesis (Stratagene, La Jolla, CA, USA) and similarly inserted into pmirGLO. SW480 and HCT116 cells were co-transfected with either the WT or MT reporter plasmid, along with *miR-7847-3p* mimic, inhibitor, or corresponding negative controls. Luciferase activity was measured following a 48-hour transfection period.

### Statistical Analysis

The study was statistically analyzed using SPSS 23.0 (IBM SPSS Corp.; Armonk, NY, USA) and GraphPad Prism 9.0 (GraphPad Software; San Diego, CA, USA). Measurements are shown as mean ± standard deviation. This study utilized G*Power 3.1.9.7 software (Heinrich Heine University Düsseldorf; Düsseldorf, Germany) for sample size and power analysis. The a priori analysis revealed that a total sample size of 172 is required with *α* = 0.05, power (1-β) of 0.90, and effect size *d* = 0.5, with the actual power exceeding 0.90. Post hoc analysis showed that with sample sizes of 105 and 122 and an effect size of *d* = 0.88, the power exceeded 0.99. The sample size is sufficient to reliably detect differences between the 2 groups. The Kaplan–Meier approach was employed to generate survival probability curves, with intergroup comparisons performed using the nonparametric log-rank test; prognostic determinants were evaluated using Cox proportional hazards modeling. Clinicopathological correlations were analyzed by chi-square test. Intergroup comparisons were performed employing Student’s *t*-test for pairwise analyses and 1-way ANOVA for multiple group comparisons. The predetermined significance criterion was *P* < .05 for all analyses.

## Results

### Analysis of the *miR-7847-3p* Expression in Colorectal Cancer and Its Correlation with Clinical Features

**Figure 1.**
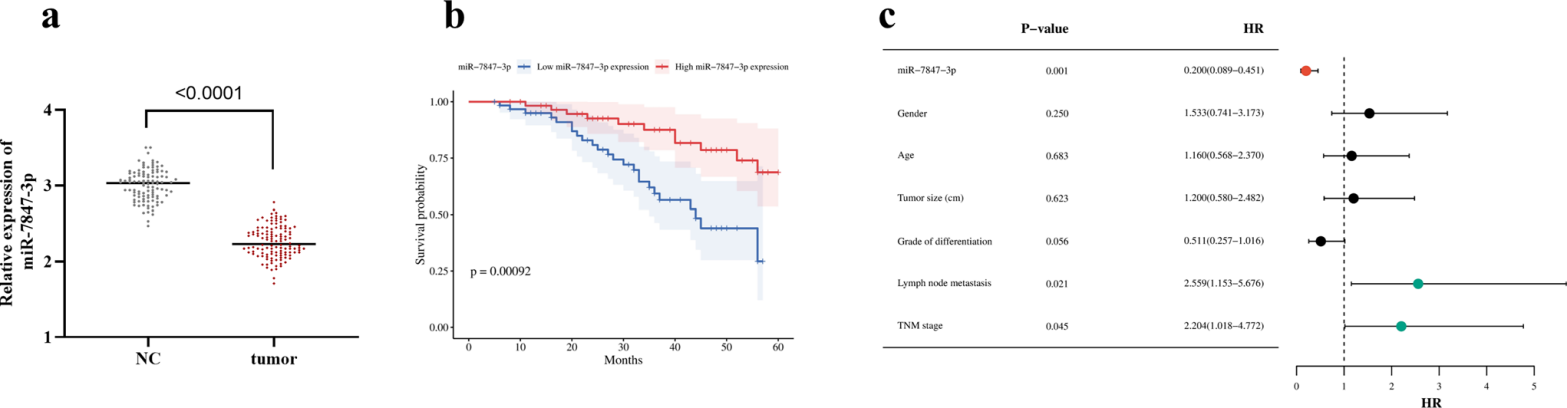
*miR-7847-3p* is downregulated in CRC and associated with poor prognosis. (a) (5) *miR-7847-3p* is expressed at a lower level in CRC tissues. (b) Relationship between *miR-7847-3p* expression level and overall survival of patients. The data was presented as mean ± SD. (c) Multifactorial Cox regression analysis of prognostic factors in CRC; HR stands for Hazard Ratio, the following value represents the 95% CI.

Tumor tissues showed a significant reduction in *miR-7847-3p* expression (*P* < .0001, Figure 1a). The *miR-7847-3p* expression showed a significant association with Tumor-node-metastasis (TNM) stage and lymph node metastasis (LNM) of CRC subjects. The proportion of participants in the low-expression group who were in TNM stage III and had LNM was higher than that in the high-expression group. No significant associations were observed between* miR-7847-3p* and participants’ gender, age, tumor size, or tumor differentiation (Table 1).

### Association Between* miR-7847-3p* Expression Levels and Clinical Prognosis of Colorectal Cancer

Kaplan–Meier curves demonstrated markedly reduced overall survival in low *miR-7847-3p* expressers relative to high expressers (Figure 1b). In addition, *miR-7847-3p* (HR = 0.200, 95% CI: 0.089-0.451, *P* = .001), LNM (HR = 2.559, 95% CI: 1.153-5.676, *P* = .021) and TNM stage (HR = 2.204, 95% CI:1.018-4.772, *P* = .045) were considered as independent prognostic factors for CRC (Figure 1c).

### Effect of* miR-7847-3p* on the Function of Colorectal Cancer Cells

**Figure 2.**
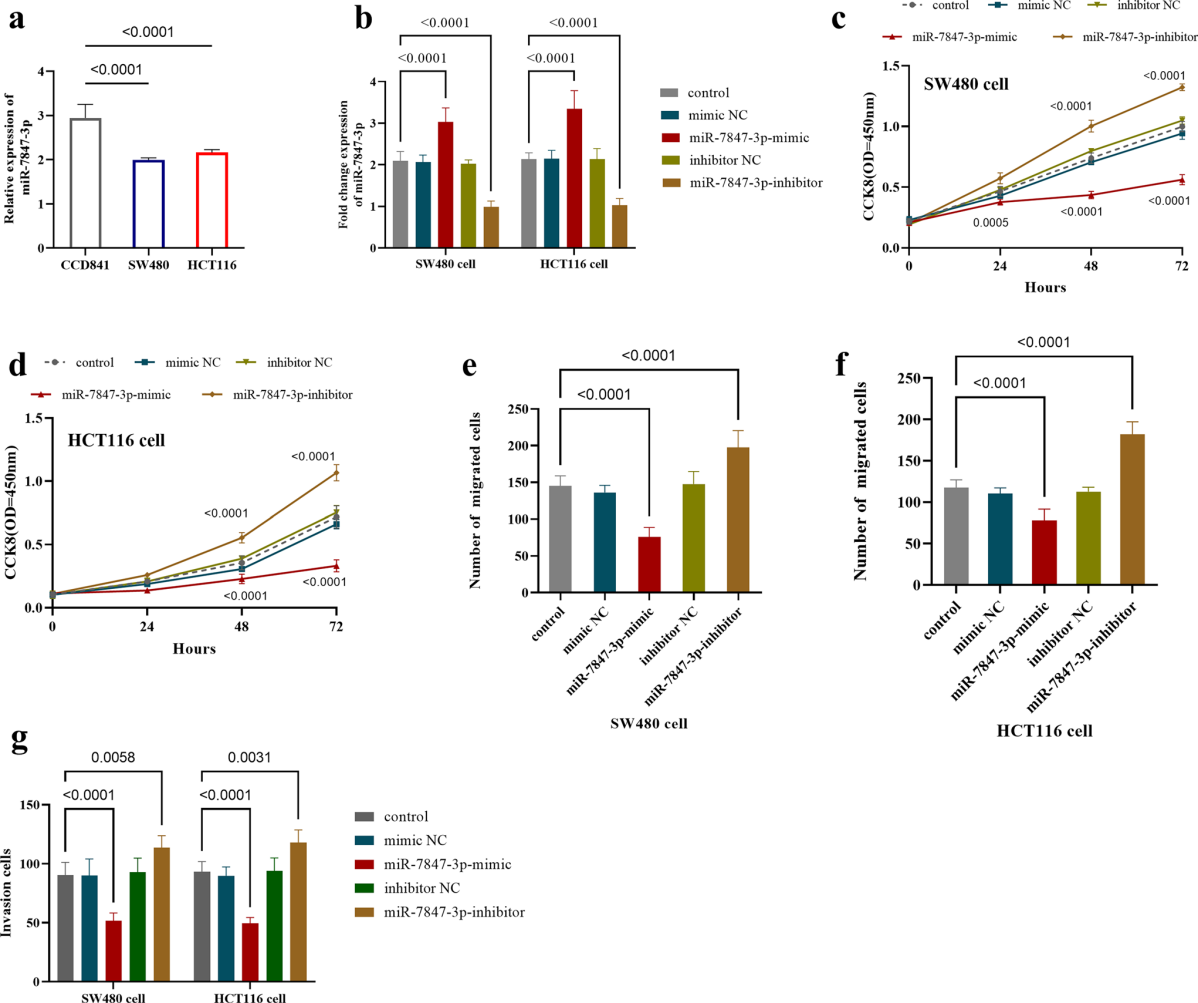
*miR-7847-3p* expression and its effect on the function of CRC cell lines. (a) *miR-7847-3p* is downregulated in CRC cell lines compared to normal cell lines. (b) Expression levels of *miR-7847-3p* after overexpression or inhibition of* miR-7847-3p* in CRC cells. (c-d) Overexpression of *miR-7847-3p* inhibited the proliferation of (c) SW480 and (d) HCT116 cells. (e-f) Overexpression of *miR-7847-3p* inhibits the migration of (e) SW480 and (f) HCT116 cells, while inhibition of its expression promotes cell migration. (g) Overexpression of *miR-7847-3p* inhibits cell invasion, while suppression of its expression promotes cell invasion in both SW480 and HCT116 cells; The data was presented as mean ± SD.

Polymerase chain reaction analysis indicated that *miR-7847-3p* expression was significantly reduced (*P* < .0001) in CRC cell lines (SW480 and HCT116 cells, Figure 2a). In both cell lines, transfection with *miR-7847-3p* mimics significantly upregulated its expression levels (*P* < .0001), while transfection with inhibitors effectively downregulated its expression (*P* < .0001, Figure 2b).

Functional assessment using CCK-8 showed that overexpressing *miR-7847-3p* reduced SW480 cell proliferation, whereas its depletion enhanced proliferative capacity (Figure 2c). This regulatory pattern was consistently observed in HCT116 cells (Figure 2d).

Transwell tests gauged *miR-7847-3p*’s impact on SW480 and HCT116 cell migration and invasion. Findings revealed that* miR-7847-3p* overexpression significantly reduced the migration of SW480 cells (*P* < .0001), while inhibition of its expression raised migrating cell numbers (*P* < .0001, Figure 2E). A similar phenomenon was observed in HCT116 cells, where *miR-7847-3p* overexpression suppressed cell migration (*P* < .0001), while *miR-7847-3p* underexpression enhanced migration (*P* < .0001, Figure 2F). In both cell lines, it was also observed that overexpression of *miR-7847-3p* reduced cell invasion capacity (*P* < .0001), while inhibition of its expression increased invasion capacity (Figure 2g).

### *AP1S1* is a downstream Target Gene of *miR-7847-3p*

**Figure 3.**
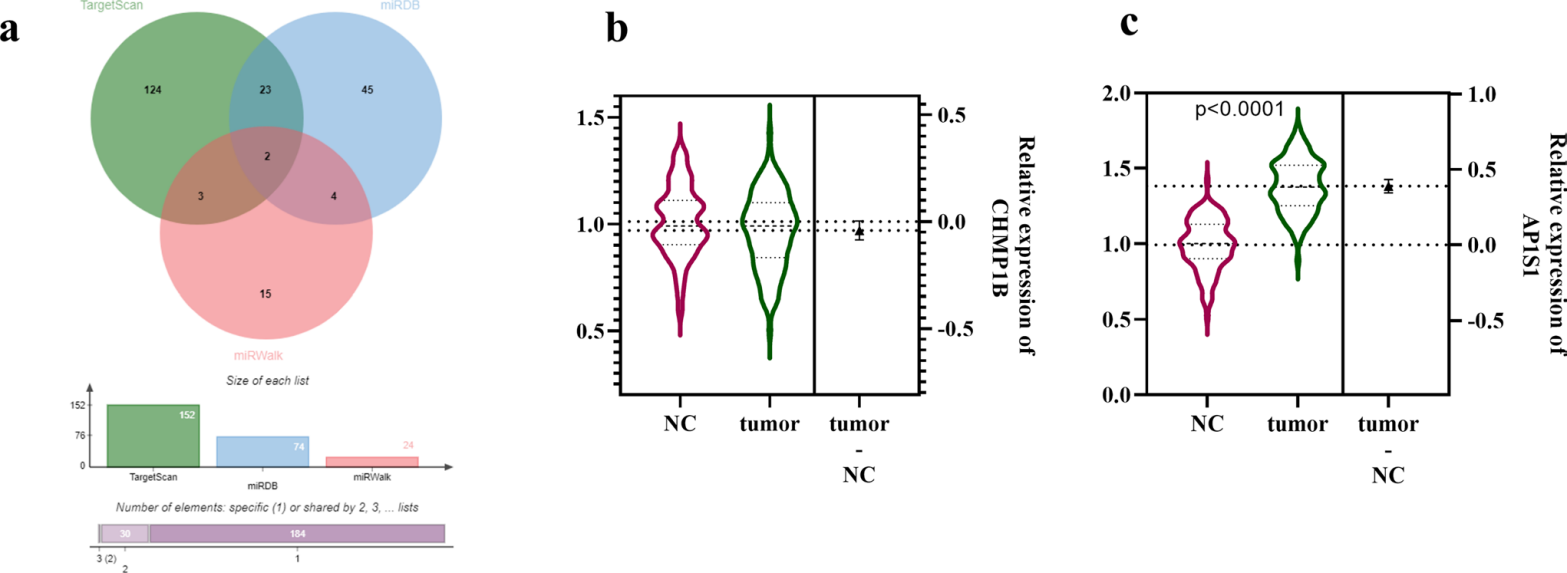
miR-7847-3p target gene screening and validation of CHMP1B and* AP1S1* expression in CRC. (a) Bioinformatics target gene prediction analysis. (b) Expression level of CHMP1B in CRC. (c) Expression validation of *AP1S1* in CRC; The data was presented as mean ± SD.

Bioinformatics analysis identified 2 potential target genes of *miR-7847-3p*, CHMPIB, and AP1S1 (Figure 3a). Subsequent expression analysis revealed that the expression level of CHMPIB did not show a significant difference between tumor tissues and adjacent non-tumor tissues (Figure 3b). In contrast, *AP1S1* exhibited markedly elevated expression in tumor tissues than in adjacent non-tumor tissues (Figure 3c).

### Validation of the Interaction Between *miR-7847-3p* and *AP1S1*

**Figure 4.**
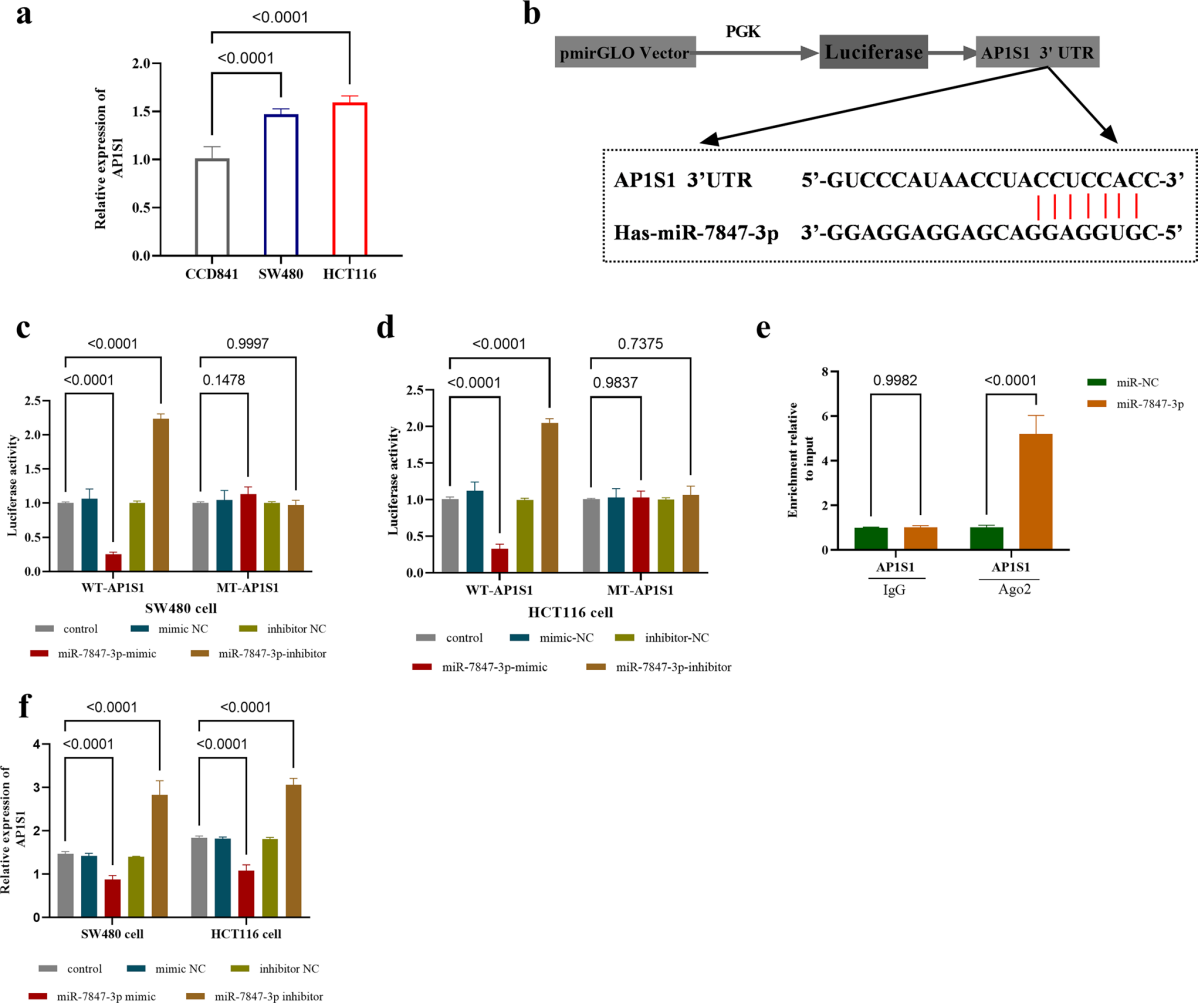
Interactions and regulatory studies of *miR-7847-3p* with *AP1S1*. (a) *AP1S1* expression in normal cells CCD841 and CRC cell lines SW480 and HCT116. (b) Binding sites between *miR-7847-3p* and *AP1S1*. (c-d) Dual-luciferase reporter assay in (c) SW480 and (d) HCT116 cells. (e) RIP assay in SW480 cells. (f) miR-7847-3 negatively regulates *AP1S1* expression in CRC cell lines (SW480 and HCT116 cells); The data were presented as mean ± SD.

Quantitative analysis of *AP1S1* expression in CRC cell lines (SW480 and HCT116) revealed that its expression level was significantly upregulated compared with normal colonic epithelial cells (*P* < .0001, Figure 4a). The binding site between *miR-7847-3p* and *AP1S1* was identified by the TargetScan online database (Figure 4b). Further dual-luciferase reporter assays showed that overexpression of *miR-7847-3p* inhibited the luciferase activity of the WT-*AP1S1* and knockdown of *miR-7847-3p* significantly promoted the luciferase activity of the WT-*AP1S1* in both SW480 and HCT116 cells, while *miR-7847-3p* had no significant effect on the luciferase activity of the mutant type (Figure 4c, d).

The RIP experiments further indicated that *miR-7847-3p* was significantly enriched in the Ago2 protein complex (*P* < .0001 compared to IgG control) and overexpression of *miR-7847-3p* increased the association between *AP1S1* and Ago2 (Figure 4e). The qPCR analysis confirmed that enforced *miR-7847-3p* expression strongly attenuated *AP1S1* levels (*P* < .0001), while knockdown of *miR-7847-3p* significantly upregulated *AP1S1* expression (*P* < .0001, Figure 4f).

## Discussion

In recent years, miRNAs have emerged as important biomarkers for CRC due to their stable presence in bodily fluids and their role in tumor regulation.^20^ Numerous studies have demonstrated that alterations in miRNA expression profiles such as miR-130b-3p and miR-129-5p are closely associated with the onset and progression of CRC.^21^^,^^22^ In addition, miRNAs such as miR-135b and miR-223 have been widely investigated as CRC diagnostic markers.^23^^,^^24^ However, their expression differences between CRC tissues and normal tissues are relatively small, which limits their application in early diagnosis. In this study, systematic analysis revealed that* miR-7847-3p* was significantly downregulated in CRC tissues, consistent with previous reports of its low expression in serum exosomes from CRC patients,^18^ and provided a new candidate biomarker for the molecular diagnosis of CRC.

Although miR-135b and miR-223 play a certain role in the diagnosis of CRC, their prognostic value is relatively limited, and there is a scarcity of related research.^25^ Clinical data analysis has revealed the significant clinical value of *miR-7847-3p* in CRC. The levels of *miR-7847-3p* in CRC tumor tissues are significantly associated with TNM stage and LNM, which are important clinical indicators for assessing the progression and prognosis of CRC. Specifically, the TNM stage remains the gold standard for therapeutic decision-making, while LNM status (N-stage) is the strongest predictor of recurrence risk and a key determinant for adjuvant chemotherapy recommendations in stage II/III cancers.^1^^,^^26^ Kaplan–Meier analysis linked low *miR-7847-3p* expression to worse 5-year survival in CRC patients, and Cox regression confirmed it as an independent poor prognostic marker. These findings suggest its potential utility in risk stratification, though prospective validation is required before considering clinical translation. Notably, this study also found that LNM and TNM stages are equally independent prognostic factors, consistent with the results of many clinical studies.^26^ Based on the findings of this study, *miR-7847-3p* may serve as an effective prognostic marker for identifying CRC patients with potential adverse outcomes.

To investigate the tumor-regulatory function and molecular mechanisms of *miR-7847-3p* in CRC, a comprehensive suite of functional characterization experiments was conducted. Cell proliferation, migration, and invasion capabilities are key indicators for assessing the malignancy of tumors: abnormal proliferation driven by dysregulation of cell cycle checkpoints is the basis of tumor development, migration capabilities mediated by cytoskeleton remodeling determine the degree of local tumor infiltration, and invasion capabilities involving degradation of extracellular matrix are directly related to the occurrence of distant tumor metastasis.^27^ The experimental results showed that overexpression of *miR-7847-3p* significantly inhibited the proliferation, migration, and invasion capabilities of CRC cells. These functional assays collectively establish *miR-7847-3p* as a tumor suppressor in CRC development.

*AP1S1*, as the core subunit of the adaptor protein complex AP-1, plays a crucial regulatory role in the endocytosis and metastasis processes of tumor cells.^28^ Studies have shown that *AP1S1* participates in tumorigenesis and progression by influencing the internalization process of receptor tyrosine kinases such as estimated glomerular filtration rate,^29^ and its abnormal expression is closely associated with the invasion and metastasis of various malignant tumors.^30^ The findings of this study further confirm that *miR-7847-3p* may exert its anti-cancer effects by targeting and suppressing the expression of *AP1S1*.

This study still has the following areas that require improvement: firstly, the limited number of clinical samples may have affected the analysis of the association between *miR-7847-3p* expression and clinical pathological features related to tumor malignancy (such as tumor size and differentiation). Secondly, due to some unforeseen factors, including inconsistent tumor engraftment rates and health issues in mice unrelated to tumor xenografts, the experimental conditions were complicated, resulting in a high failure rate in animal model construction. Thirdly, while it has been confirmed that *miR-7847-3p* exerts its anticancer effects by targeting *AP1S1*, the specific molecular mechanisms of *AP1S1* in CRC require further exploration. Fourth, although the study design has attempted to control for confounding factors, there may still be other unidentified or unmeasured confounders that could influence the expression of *miR-7847-3p* and its relationship with the clinicopathological characteristics of CRC. Finally, although the variation in the severity of patients’ conditions leads to the inability to standardize postoperative treatment plans, postoperative treatment may influence the prognosis, a factor that was not taken into consideration in this study. These limitations will be the focus of future research.

This study reveals the significant role of *miR-7847-3p* in CRC, with its low expression being associated with poor prognosis in CRC patients.* miR-7847-3p* plays an antitumor effect in CRC by inhibiting cell proliferation, migration, and invasion of CRC cells. Furthermore, this study also confirmed that *miR-7847-3p* can predict patient prognosis and regulate cellular processes by targeting *AP1S1*. This discovery provides a novel biomarker for the molecular diagnosis and prognostic assessment of CRC, with potential clinical applications such as assisting in risk stratification and guiding the formulation of personalized treatment plans. Furthermore, the elucidation of the *miR-7847-3p*-*AP1S1* axis provides direction for in-depth exploration of the pathogenesis of CRC and the development of novel therapeutic targets. Future research should expand the sample size to validate the prognostic efficacy of *miR-7847-3p*, delve deeper into its molecular mechanisms with* AP1S1*, and promote its clinical translation and application.

## Figures and Tables

**Table 1.** Correlation of *miR-7847-3p* with Clinicopathologic Characteristics of Patients

	Cases	*miR-7847-3p* Expression		*P*
		Low	High	
Total	122	62	60	
Age (years)				.966
<55	47	24	23	
≥55	75	38	37	
Gender				.717
Male	61	30	31	
Female	61	32	29	
TNM stage				**.020**
I-II	79	34	45	
III	43	28	15	
Grade of differentiation				.80
Well-moderate	78	35	43	
Poor	44	27	17	
Tumor size (cm)				.064
<4	69	30	39	
≥4	53	32	21	
Lymph node metastasis				**<.001**
No	81	30	49	
Yes	41	30	11	

Data are expressed as a number of cases. The correlation between *miR-7847-3p* expression (low/high) and clinicopathologic parameters was assessed using a chi-square test. Bold *P*-values indicate statistical significance (*P* < 0.05).

## Data Availability

The datasets used and/or analyzed during the current study are available from the corresponding author on reasonable request.
